# A Photoaging Intervention Delivered to Adolescents in Secondary Schools: A Feasibility Study

**DOI:** 10.1155/2022/9434176

**Published:** 2022-07-19

**Authors:** Bronwen M. McNoe, Kate C. Morgaine, Anthony I. Reeder, Ella Iosua

**Affiliations:** ^1^Social & Behavioural Research Unit, Department of Preventive & Social Medicine, Dunedin School of Medicine, University of Otago, Dunedin, New Zealand; ^2^Department of Preventive & Social Medicine, Dunedin School of Medicine, University of Otago, Dunedin, New Zealand; ^3^Centre for Biostatistics, Dunedin School of Medicine, University of Otago, Dunedin, New Zealand

## Abstract

Excessive exposure to ultraviolet radiation during adolescence can have a lasting effect on long-term skin cancer risk. Skin cancer prevention interventions for adolescents have been less commonly investigated than those for children and adults. The study objectives were to develop and evaluate the feasibility of a secondary school-based appearance focused intervention, including the development and testing of protocols and instruments, as a resource module that could be efficiently integrated into the secondary school science curriculum. This longitudinal study was conducted with a convenience sample of 38 13–14 year-old students attending one New Zealand (NZ) urban secondary school. The recruitment rate was excellent with only one student not participating because of parental concern. In terms of the implementation practicality, the intervention, as it stands, was extremely resource intensive, involving four research staff to deliver. This will not work if delivered in a classroom setting by a single teacher. However, the intervention was well received by students, so it shows promise if a less resource intensive version could be produced. The acceptability of the intervention with the students was good with the majority (61%) having no suggestions for improvements. Suggested improvements were minor and could be easily addressed.

## 1. Introduction

Skin cancer is a substantial public health issue globally [1]. Understanding the aetiology of this disease is well advanced, and there is consensus in the scientific literature that exposure to ultraviolet radiation (UVR), primarily from the Sun, is the major modifiable risk factor [2]. The best opportunities for reducing the frequency of skin cancer are in primary prevention interventions that reduce harmful sun exposure, thereby reducing DNA damage and potential skin cancer development. The available evidence suggests that young people are an important group on which to focus efforts, and adolescents, in particular, have been neglected in sun protection initiatives [3].

Adolescents' psychological and behavioral characteristics, including their attitudes to, knowledge of and beliefs about skin cancer prevention, sun protection behavior, and sun protection practices, are key in developing personal skills [4]. Educational settings are potentially important for the development of health literacy on skin cancer control. It is essential that students should be not only instructed in the need to use sun protection, but also educated about the reasons why it is necessary, thereby developing the knowledge that should help them protect themselves throughout life [5]. Skin cancer prevention interventions for adolescents have been less commonly investigated than those for children and adults. A recent systematic review identified that appearance-based interventions may be beneficial for this age group [3]. The study objectives were to develop and evaluate the feasibility of a secondary school-based appearance focused intervention, including the development and testing of protocols and instruments, as a resource module that could be efficiently integrated into the secondary school science curriculum.

## 2. Methods

### 2.1. Type of Study

This is a longitudinal feasibility study [6].

### 2.2. Study Population

The study population was a convenience sample of two classes of year 9 (aged 13-14 years old) adolescents based at one urban New Zealand (NZ) secondary school. As this was a feasibility study, there was no intention to recruit a representative sample, there was no control group, and the study was not powered to measure or assess effectiveness.

### 2.3. Research Questions

- What are the scientific factors necessary for an appearance-based intervention delivered in secondary schools for students? Factors considered included recruitment rate, intervention adherence, intervention implementation practicality, and intervention acceptability for students.
- What are the management processes for a potential future randomized controlled trial (RCT) delivered in secondary schools of an appearance-based intervention intended to reduce UVR exposure of students?

### 2.4. Recruitment

An exploratory conversation with the head of junior science at the trial school was used to gauge interest in the project; this was found to be affirmative. A formal invitation was then extended to the school principal asking if they would be prepared to allow the students to participate in the study within the school setting, to which they consented. The school was reimbursed at a rate of NZ$10 per student to compensate for the burden of additional work for staff. The timeframe (December in southern hemisphere summer) was purposively chosen as that is when senior students have left the school for external exams, the main curricula for the year are finished, and teachers are actively engaged in keeping junior students occupied. The lead author visited the school, spoke with staff about the project, and provided recruitment materials for students and parents/caregivers. These included separate information sheets and consent forms for parents and students using age-appropriate language. In addition, written copies of the questionnaire (described below) were included. The instructions provided to staff were that the questionnaire should be completed in class time, one week prior to the intervention. Those students for whom either their parent/caregiver or they themselves did not provide consent were given alternative activities to do, while their classmates completed the intervention.

### 2.5. Instrument

The questionnaire used was the Sun Exposure and Protection Index (SEPI) (part II) [7] modified slightly for the NZ environment. The SEPI contains four questions relating to the propensity to increase sun protection (including the use of sunscreen, clothing, a sun-protective hat, and shade) and one question about giving up sunbathing, producing five outcome measures. Students completed the SEPI at baseline during class-time one week prior to the research team coming into the school to deliver the intervention and then again immediately following the intervention.

### 2.6. The Intervention

Two theoretical models were important in the development of this intervention: the Health Action Process Approach (HAPA) [8] and Dissonance Theory [9]. The HAPA was conceived to address the gap between intention and behavior, commonly observed in adolescents. Perceived self-efficacy to engage in sun protective activities is vital, and so risk perceptions (for example, of photoaging) play a role in the motivation phase of the HAPA. Similarly, outcome expectations are important when individuals balance the pros and cons of the consequences of UVR exposure or the adoption of sun protection behaviors. Adolescents need to be motivated to perform sun protective behaviors; in the case of this specific intervention by the use of photoaging techniques, with the aim that this motivation will be translated into positive behavior change. Dissonance theory postulates that when attitudes and behavior conflict, the person will experience physical and psychological discomfort that leads them either to change their attitudes to fit their behavior or vice versa. For example, if adolescents are exposing themselves to UVR to obtain a tan, and appearance is important to them, then it follows that observing UVR related skin damage should alter their sun protection behavior [10].

### 2.7. Intervention Content

The intervention consisted of UV (ultraviolet) photographs, a PowerPoint presentation, and other UVR “experimental” activities. The UV camera, software, and associated equipment included Profect® Studio and Ultra II software owned by the Health Promotion Agency and loaned to the researchers at no cost. The UV camera was mounted in a light controlled “tent” with a seat and chin rest for participants in order to limit head movement. Each photographic session took approximately three minutes. Three black and white photographs were taken of each student: the first under “white light,” the second using a UV filter that displays signs of sun damage not visible to the naked eye, and the third using the same UV filter but with sunscreen applied to the participant. This demonstrated the comprehensiveness of sunscreen coverage and, by blackening the protected areas, visualizes the effect that sunscreen has in blocking UVR. The visualization of sunscreen coverage would not be as effective on students with dark or very dark skin; however, this was not the case for any of the study cohort. A health promoter then viewed these three images with each student and talked about any evidence of skin damage and what the student needed to be doing in terms of sun protection. This conversation was not scripted but tailored to the individual student (for example, their skin type) and their interests (for example, the student was asked “what summer sports do you play?”). A PowerPoint presentation about the effect of UVR exposure on human skin, in particular, on light colored skin types, was also delivered to students. The slides were designed to emphasize the effect that UVR has on appearance and had two main “take home” messages: “you cannot see or feel UVR” and “excessive UVR results in photoaging of the skin.” Skin cancer was intentionally not mentioned. The individual slides covered relevant issues around electromagnetic radiation, including:

  The types of UV light and the effects that different wavelength bands have on human skin (e.g., burning and tanning);
  How UVR affects skin, the influence of melanin and skin type, the photoaging effect of UVA and the effects of lifetime cumulative sun exposure;
  How UV light is invisible to the human eye but can be seen by other animals, and the visual effect of UV light on skin demonstrable by using UV photography;
  The Ultraviolet Index (UVI), factors determining UVR level, why there are high summer UVR levels in NZ;
  When and how to protect ourselves from UVR (including how sunscreen works, and the issues around vitamin D, skin color, and social norms (for example, regarding sunbed use);
  A video showing faces exposed to UV light.

The presentation was approximately one hour in duration and delivered to the students in a classroom setting by a trained health promoter. As the photographic feedback component of the intervention was delivered to one student at a time, other activities had to be included to keep the remaining students occupied. The targeted message from these interactive activities was to demonstrate that you cannot see or feel UV light, but that it is real, and to demonstrate the effectiveness of some sun protection strategies. A trained health promoter delivered and supervised this component of the intervention. These activities were as follows:

- Viewing how tonic water fluoresces under UV light, whereas tap water does not. Students were given “black light” torches that emit UV light and jars containing tonic water and tap water so they could see this for themselves. They were then able to place sunglasses between the light source and the tonic water to see the effect sunglasses have on blocking the transmission of UVR.
- Investigating the application of UV light in real life situations: students were provided with physical examples of products that use this technology and asked to make comparisons on what they could see under white light and UV light. Examples of the items included a passport and the paper money of various countries.
- Observing how UV sensitive beads are white when there is no UVR present but become colorful when exposed to UVR. Students made a bracelet with white UV beads and then went outside to see the beads change color in response to UVR exposure. The effect of window glass on the UV beads was also assessed.
- Students were given a visual demonstration of how the uv2day app works [11]. This is a NZ designed product that displays the UVI at any particular time of day and on any day of the year at the exact location of the user.

### 2.8. Intervention Delivery

The activities were divided into two sessions of approximately 60 minutes each. Session 1 was the PowerPoint presentation, and Session 2 included the UV photography, feedback, and filler activities. The students were divided into two groups with approximately 20 in each. These groups received either Session 1 or Session 2 in the first hour, followed by morning tea, and then the alternative session, followed by lunch. The two groups were subsequently brought back together for a “wrap-up” session in which the student evaluation and follow-up SEPI (part II) was conducted. The purpose of the student evaluation was explained to students, emphasizing the value of genuine feedback, even if it was negative, as providing an opportunity to improve the intervention for other students. Pens and tubes of SPF 30+ sunscreen were given to students in appreciation for their participation in the project.

### 2.9. Data Analysis

All quantitative analyses were performed in Stata [12]. Questionnaire responses were reported using descriptive analysis. Sociodemographic characteristics and each of the outcome measures were reported as either a percentage or mean at baseline and follow-up depending on whether it was a categorical or continuous variable, respectively. An additive score was created using SEPI (part II) guidelines, [7] ranging in value from 0 to 20 with “a higher score reflecting a low propensity to increase sun protection” [7]. A paired *t*-test was used to explore changes in the SEPI (part II) score between baseline and follow-up.

## 3. Results

Of 49 students in the two Year 9 classes, consent from both parents and students was available for 48 to participate in the study and complete the SEPI one week prior to the intervention. On the day of the intervention, 38 of these students were present at school, attended the intervention, and completed the evaluation. Of the 38 students that completed the follow-up, 16 (42%) were 13 years of age and 22 (58%) 14 years, and just over half (*n* = 20, 53%) were female. More than one ethnicity could be reported with most students (*n* = 36, 95%) reporting that they were NZ European, six (16%) Māori, and three (8%) another ethnicity. Skin type was not recorded.

### 3.1. Sun Protection Attitudes at Follow-Up Compared to Baseline

The range of SEPI (part II) scores was 1–16 at baseline and 0–16 at follow-up. The mean score at baseline was 8.10 (standard deviation (SD) 4.42) and immediately following the intervention, it was slightly reduced and more protective at 7.63 (SD 4.60). The mean difference was 0.47 (95% CI −0.50, 1.44) suggesting that the intervention had no to minimal effect on the SEPI (part II) score. A feasibility study, however, is not designed to have the capacity to estimate effects with precision, and therefore, these results should only be used descriptively and to provide parameter estimates to inform a sample size calculation for a possible future RCT.

### 3.2. Research Team Evaluation

The UV photographic intervention was well received by students. There were, however, a number of issues with managing the UV photography. It took more than one hour to set up (and a similar time to take down) the photographic equipment and the tent, including the time required to adjust the equipment to get the best resolution. The quality of the photographs was not high, and although they were compelling for students who had apparent sun damage, this was less so for others. A number of teachers came forward and had their photograph taken and appeared to be more engaged than the students although a number of students did say in the evaluation that they liked the UV photographs. Students wanted to know things like “what are the dark spots?” “Have I got skin cancer?” “I have spent lots of time in the Sun, why do I not have damage?” And “Is my skin bad compared to others?”

### 3.3. Experimental Activities

The students enjoyed seeing the real-life applications of UV light, particularly in relation to the passports and paper money. The tent blocked out all natural light and was very good at maximizing the visual appearance of UV. However, the maximum number of students that could be accommodated in the tent at one time was only five. With respect to the UV beads, initial concerns raised by health promoters that these might be considered “too juvenile” for this adolescent group were not confirmed as the students enjoyed this activity. The demonstration of the uv2day app ran smoothly, but the health promoter noted that several students did not have a smartphone. This may single them out as economically disadvantaged and subsequently may, therefore, not be an appropriate activity for a school setting. Furthermore, at the “wrap-up” session, only three students reported that they would download the app. Overall, there was too much time spent waiting around during the experimental session. The students appreciated the small gifts that included food and low-cost pens and sunscreen.

### 3.4. PowerPoint Presentation

Each of the presentation slides was evaluated, and modifications were made as suggested by the health promoter who delivered the presentation. Some of the presentation content was not directly relevant but was included in order to engage student interest and provide examples of “sun protection messages.” For example, when discussing vitamin D, the health promoter talked about the Vietnam War, with respect to how Vietnamese children spent time underground to reduce the risks from warfare and subsequently developed rickets. This led to quite a long discussion with the class. Students responded particularly well to video clips. These responses tended to be gendered, with girls particularly engaged with “cuteness” type imagery, whereas boys were more interested in sporty images. The health promoter felt that students were most engaged with the imagery of local scenes they recognized and local sports stars, as well as those that were age appropriate, that is, images of teenagers rather than young children. The health promoter considered that the class to which the presentation was delivered following the practical session was less engaged than the class to which the presentation was delivered first, potentially due to fatigue.

### 3.5. Student Evaluation

At the conclusion of the session, students were asked to recall two facts from the intervention. Interestingly, seven students mentioned skin cancer although this was deliberately not mentioned during the intervention. Only three of the 38 students gave factually incorrect information: one confused UVR with temperature, one gave an incorrect sun protection message, and one an incorrect skin cancer fact. Disappointingly, given the intended focus of the intervention, only one student specifically mentioned appearance. Most of the facts recalled were related to the sun protection messages and properties of UVR. Students reported that what they most liked about the intervention were the UV photographs and gaining new knowledge. The most commonly reported “dislike” was the PowerPoint presentation, which six students reported as being either “too long” or “boring.”

Students were also asked if anything could be done to improve the intervention. Most students regarded it very positively and did not have suggestions for improvement (*n* = 30). Three students said it would be good to have better quality photographs and one student that they would like to have been able to take the photo away afterwards. A few students wanted either a more interactive (*n* = 2) or “fun” (*n* = 1) session, and one suggested that the slideshow should have more musical accompaniment.

### 3.6. Staff Feedback

The school head of science was able to provide insight into how core science is taught at this level in many secondary schools in NZ, being distinctly broken into its component parts of physics, biology, and chemistry. The intervention in its current form crosses all three of these disciplines. For example, the structure of UVR would fit into the physics curriculum, sunscreen the chemistry curriculum, and the way animals adapt to their environment, the biology curriculum. She suggested that the intervention may be better placed in the physical education and health curriculum or that it be targeted at Year 8 students (aged 12 years) who receive the science curriculum as one core subject.

## 4. Discussion

The findings from this study relate to the feasibility of conducting an appearance focused intervention in a secondary school setting in NZ among Year 9 students. It was intended that this intervention should be appropriate for integration within the secondary school science curriculum and delivered by the classroom teacher.

The secondary school environment is an ideal setting for interventions because it potentially provides an opportunity to engage simultaneously with large groups of young people. Equally important is the timeliness, given that this is the life stage when children move into adolescence and form their attitudes and opinions with respect to sun protection, tanning, and UVR exposure [13]. Cancer education that is integrated into the curriculum and presented in such a way that it encourages student interaction is potentially the most effective way of promoting behavior change [14]. However, secondary schools are a challenging environment for those advocating for skin cancer prevention, and a number of challenges were experienced with implementing the intervention described here. Sun protection policies, practices, and curriculum content are, currently, not a priority for NZ secondary schools [15]. There are no specific Ministry of Education regulations about skin cancer prevention strategies or education. The secondary curriculum is already overcrowded, with teachers struggling to accommodate all they are required to teach, providing limited opportunities for covering additional noncompulsory topics, such as sun protection. The aspirational goal in developing this intervention, should it prove to be effective, was to provide secondary school teachers with a good quality resource that met specific curricula requirements to use in their everyday teaching, rather than asking them to do an “additional activity.” When the intervention was conceived, the research team believed that it would be best placed in the science curriculum. Skin cancer prevention is naturally placed in science, [16] and science in the NZ curriculum is a compulsory subject for all students until year 10. By addressing specific requirements of the science curriculum for Years 9-10, it would be attractive for staff to adopt without increasing their workload. Essentially, UVR and sun protection could be used as practical examples to deliver curricular requirements. However, the research team had not appreciated, until discussions with staff at the intervention school, that although science is still one curriculum topic at Years 9-10, operationally, within many secondary schools, it is now separated and taught in its component parts. The intervention as it currently stands could not be delivered neatly in that environment. One alternative would be to target the physical activity and health curriculum, but there it would be competing with other health concerns, some of which have much more immediate consequences among youth than skin cancer. The other alternative would be to combine sun protection with other health concerns; for example, appearance-based age progression technology has been used as a smoke free intervention [17].

A panel of international skin cancer prevention experts thought that interventions for the adolescent demographic should use immediate outcomes, such as skin damage and photoaging rather than the delayed outcomes like skin cancer [18]. However, they recommended that skin cancer should still be referenced, because fear remains a powerful motivator [18]. Although the emphasis in the current intervention was placed on the effect of UVR on appearance and that you cannot see or feel UVR, these were not at the forefront of students' minds when they responded to being asked to report any two facts that they took away from the intervention. Interestingly, although skin cancer was deliberately not mentioned during the intervention, a number of students did mention skin cancer in the facts learned.

There was some evidence that those students who received the intervention in the second session may have been fatigued, so it may be better to have this included in the first session for all students. This would be challenging in terms of dealing with the number of students in a class as the intervention itself is one-on-one, and so, only a certain number of students can be photographed within a specific time slot. Potentially, this could be resolved by having separate sessions on different days. One student did suggest that copies of their image should be provided to them, and this was also mentioned by a number of students during the intervention delivery. As the students were under 16 years, however, we believed that it would be much more difficult to obtain ethical approval because these photographs clearly identify the study participants and may leave students vulnerable to peer pressure to share the image and potential teasing. Body image has a substantial impact on the way that young people interact with their peers [19] and is reportedly one of the most common targets for bullying among students in school settings [19, 20]. Both males and females who are teased about their appearance during adolescence have a poorer body image or higher body dissatisfaction than their peers [19]. Although scientific evidence suggests that raising appearance concerns could positively impact sun protection behavior, conversely, this could also potentially have a negative effect on self-esteem and body image [21]. A promising intervention using smart phone technology to artificially age “selfies” in a group setting is also potentially open to the issues of teasing by peers [20]. Given adolescents are reliant on technology, interventions that employ electronic technology, such as smart phone apps, should be considered for skin cancer prevention in this cohort. However, it became apparent that the use of smart phones for this age group in a secondary setting is not appropriate, given the proportion of students that lacked access to this technology. Most of the other experimental activities were well received, in particular, the examples of how UVR is used in real life and the UV beads.

One of the limitations of this intervention was that it was delivered to two year groups in a single school. Evidence suggests that sun protection attitudes and behaviors deteriorate as adolescents age [22]. The outcome instrument (SEPI) has been developed and used internationally to measure behavior change in sun protection but had not previously been tested in a NZ environment or with adolescents. Our findings indicate that it was comprehensible, acceptable, and practical to administer among this population in a school setting. However, the outcome measures are self-reported behaviors, in some cases over the past 12 months, which raises the potential for both social desirability and recall biases.

## Data Availability

There are no publicly available data.
